# Distinct NK cell dynamics in SARIFA positive colorectal cancer patients indicate persistent patient-intrinsic immune signatures after tumor resection

**DOI:** 10.1038/s41598-026-63238-z

**Published:** 2026-07-29

**Authors:** Florian Sommer, Lena Anthuber, Bianca Grosser, Phillip Löhr, Andreas Rank, Eva-Maria Allmendinger, Lisa Siebenhüter, Nic G. Reitsam, Bruno Märkl, Johanna Waidhauser

**Affiliations:** 1 Department of General, Visceral, and Transplant Surgery, University of Augsburg, Augsburg, Germany; 2 Institute of Pathology, School of Medicine and Health, Technical University of Munich, Munich, Germany; 3 Hematology and Oncology, Faculty of Medicine, University of Augsburg, Augsburg, Germany; 4 Pathology, Faculty of Medicine, University of Augsburg, Augsburg, Germany; 5 Bavarian Cancer Research Center (BZKF), Augsburg, Germany; 6 Comprehensive Cancer Center Alliance WERA, Augsburg, Germany

**Keywords:** Biomarkers, Cancer, Immunology, Oncology

## Abstract

The recently introduced H&E-based biomarker SARIFA (Stroma AReactive Invasion Front Areas) is defined by a direct tumor-adipocyte interaction at the invasion front and is highly predictive for worse outcome in colorectal cancer (CRC). Further analysis revealed an altered lipid metabolism and an immune dysregulation in SARIFA-positive cancers, specifically reduced natural killer (NK) cells. In this study, we investigated whether these lower NK cells in SARIFA-positive patients persist after tumor resection, indicating a patient-intrinsic factor that might contribute to the development of SARIFA-positive tumors. Flow cytometric analysis of NK cells of CRC patients was performed before, 7–10 days and 6 months after resection of the tumors. Using multiplex ELISA (enzyme-linked immunosorbent assay), various inflammatory mediators were examined. 28 SARIFA-negative, 12 SARIFA-positive patients and 27 matched healthy individuals were included. Preoperative NK cell values were previously published and showed significantly lower values for SARIFA-positive patients. Postoperatively NK cells and all their subsets declined in SARIFA-negative patients. Regarding the 6 months follow-up measurements, SARIFA-negative patients showed increasing values of NK cells reaching the initial level, while lower NK-cell levels persisted in SARIFA-positive patients. Multiplex ELISA of inflammatory mediators revealed a postoperative increase of IL5, IL6 and IL8 but no differences between SARIFA-positive and -negative patients. Postoperative measurements of NK cells and different subsets showed a persisting difference between SARIFA-positive and SARIFA-negative patients six months after surgery, which may indicate that patient-intrinsic immunological characteristics are associated with the development of the more aggressive SARIFA-positive tumors.

**Supplementary Information:**

The online version contains supplementary material available at 10.1038/s41598-026-63238-z.

## Introduction

Accurate prediction of tumor behavior and progression is essential in clinical oncology. Beyond classical histopathological features such as grading, nodal status, lymphovascular invasion and tumor budding, immunologic components of the tumor microenvironment (TME), including regulatory T cells and tumor-associated macrophages, have demonstrated prognostic significance in CRC^1–3^. Furthermore, CRCs not only cause a remodeling of their local microenvironment but also induce reduced numbers of B-cells, T-cells, NK cells and several of their subsets in peripheral blood^4^.

Recently, Stroma Areactive Invasion Front Areas (SARIFA) has been identified as a novel adverse prognostic biomarker in colorectal, pancreatic and gastric cancers^5–8^. SARIFA denotes the direct interface between adipocytes and tumor cells at the invasive front in the absence of stromal activation, delineating a distinct microenvironmental niche associated with a more aggressive course of disease^5–7^. SARIFA positivity has been repeatedly linked to adverse clinicopathological characteristics and poor patient outcomes across several colorectal cancer (CRC) cohorts^5,9–12^.

When focusing on the immune microenvironment, SARIFA positivity is associated with a marked reduction in CD3^+^ T-cell and M1 macrophage densities, coupled with an enrichment of M2 macrophages, indicating an immunosuppressive microenvironment conducive to tumor progression^5^. SARIFA positive tumors appear to represent an intrinsically aggressive and potentially lipid-driven tumor biology, rather than merely reflecting the systemic effects of increased adiposity or obesity in the patient^13^. Spatial transcriptomic analyses have revealed a concomitant upregulation of adipocyte differentiation and lipid metabolism-associated genes such as *FABP4*, indicating that metabolic reprogramming and immune modulation, rather than purely genomic alterations, may underlie the aggressive phenotype of SARIFA positive tumors^8,14^. Recent work by our study group reported a significant reduction in circulating NK cells and their subsets in SARIFA positive CRC cases^15^.

Natural killer (NK) cells, as key effectors of innate immunity, play a pivotal role in the immunosurveillance and immunomodulation of CRC. NK cells exert potent cytotoxic activity through the release of lytic granules, engagement of death receptors, and recruitment of other immune cells via cytokine secretion, all independent of prior antigen presentation^16^. Clinically, a high intratumoral NK cell density correlates with improved prognosis and clinical outcomes in various solid tumors^17^.

Regarding the interplay between adipocytes and NK cells, several interactions are known. For example, adipocytes (especially cancer-associated adipocytes, CAAs) and tumor cells secrete interleukins - primarily IL-6 (often also IL-8/IL-1) and adipokines such as leptin - which reprogram the tumor microenvironment via JAK/STAT3 signaling^18^. IL-6 and IL-8, in turn, both from tumor cells and adipocytes, activate STAT3 in NK cells, leading to downregulation of activating receptors (e.g., NKG2D, NKp30), reduced granzyme/perforin expression, and thus impaired NK cytotoxicity. This effect has been demonstrated experimentally in patient samples, co-culture assays, and mouse models^19^. Adipocyte signals (e.g., IL-6, leptin) also alter the metabolic state and immune cell composition (e.g., shift in NK subsets), which can further weaken NK function^20^.

However, despite these insights, the directionality of this interaction remains unclear. It is currently unresolved whether SARIFA constitutes a primary driver that actively suppresses NK cell abundance and function through its adipocyte-dominated microenvironment, or whether a pre-existing reduction in NK cell-mediated immunosurveillance confers a selective advantage that facilitates the emergence of a more aggressive, SARIFA-positive tumor phenotype. Disentangling this causal relationship is essential to understand the biological significance of SARIFA and its role in tumor-immune-metabolic crosstalk in CRC. This study aims at evaluating the postoperative course and NK cell dynamics in SARIFA positive and SARIFA negative patients.

## Materials and methods

### Study design and patient inclusion

This study was conducted at University Hospital Augsburg over a two-year period from December 2018 to November 2020. 40 Patients were enrolled as part of a prospective colorectal cancer (CRC) cohort, for which previous investigations have also been published^4,15,21^. The present evaluation represents a post hoc analysis of this cohort. All included patients underwent surgical treatment without receiving neoadjuvant therapy. Surgery was performed according to established guidelines for UICC tumor stages I–III^22^.

To minimize confounding factors influencing immune function, patients with acute or chronic infections, inherited or acquired immunodeficiencies, autoimmune disorders, or concomitant malignancies were excluded from the study. Patients with stage IV disease that was pretherapeutically inapparent were likewise excluded from evaluation.

A total of 27 age matched healthy individuals served as the reference cohort. These participants were blood donors recruited from the blood bank of the University Hospital Augsburg (Bavaria, Germany). Patient demographic and tumor characteristics are depicted in Table 1.

### Ethical considerations

The study was approved by the ethics committee of Ludwig Maximilian University of Munich, the relevant ethics committee for our institution (Reference: Project No. 18–726) and conducted in accordance with the principles of the Declaration of Helsinki at University Hospital Augsburg, Germany. Prior to study inclusion, all patients provided written informed consent.

### Definition of SARIFA

**Fig. 1:**
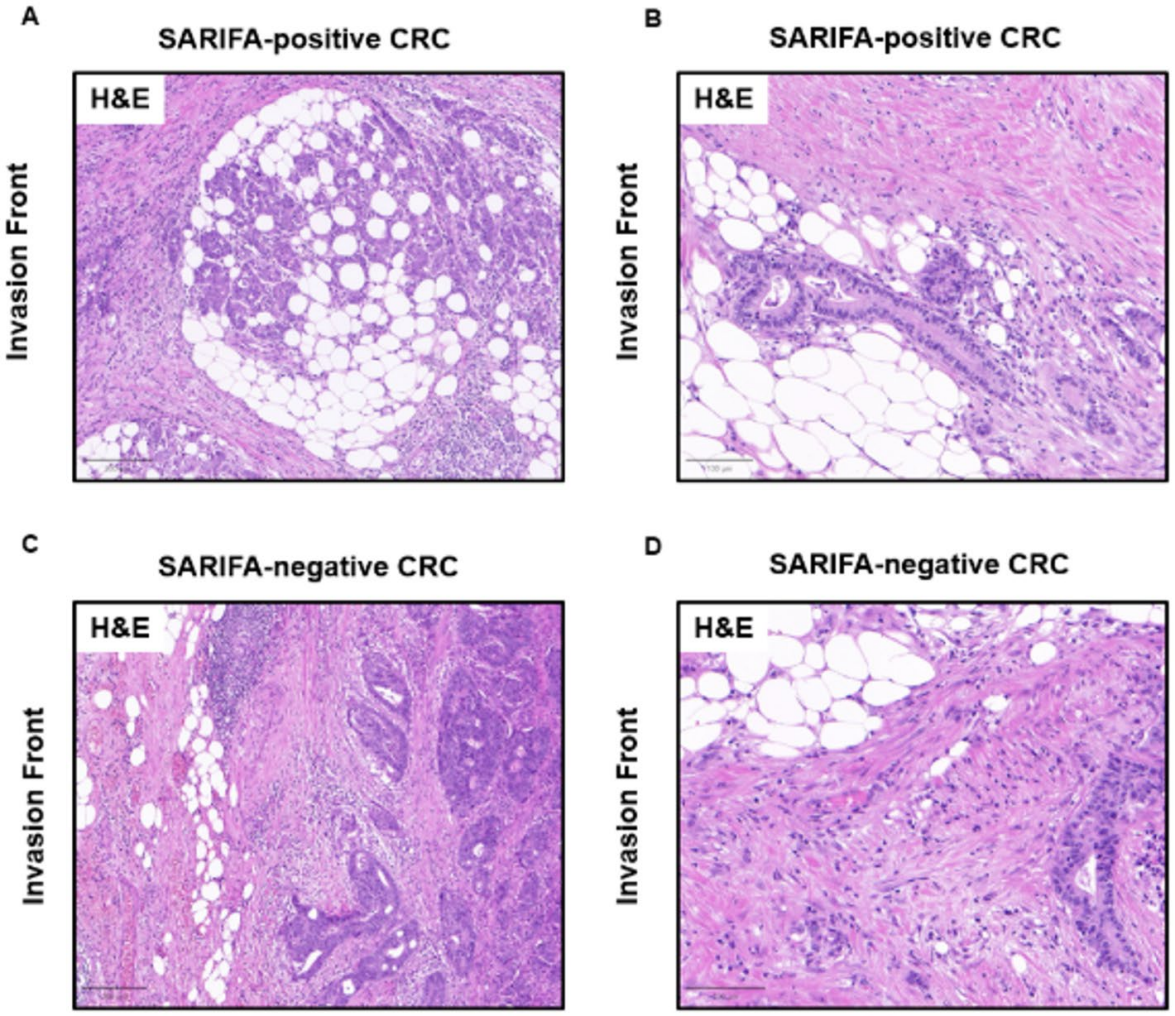
Representative H&E-stained tumor sections of SARIFA positive (**A** and **B**) with direct contact between tumor cells and adipocytes at the invasion front and SARIFA negative (**C** and **D**) CRC samples with a pronounced stromal reaction at the invasion front, preventing a direct-tumor adipocyte interaction. Scale bar = 200 μm in **A** and **C**, and 100 μm in **B** and **D**. CRC, colorectal cancer; H&E, hematoxylin and eosin; SARIFA, Stroma AReactive Invasion Front ARea.

SARIFA status was established for this patient cohort for a previous publication for which we now present updated and additional data^15^. SARIFA status was assessed across all available tumor slide sections, prioritizing those with the greatest depth of invasion. Following our previous publication on SARIFA, SARIFA was defined as a region at the tumor invasion front (IF) where at least one tumor gland, or a cluster of at least five tumor cells, is directly adjacent to adipocytes, with no intervening inflammatory infiltrate or desmoplastic stromal response on standard hematoxylin and eosin (H&E) staining^7^. The stromal reaction could include features such as collagen deposition, reactive histiocytes, or fibroblastic proliferation. The presence of even a single SARIFA, such as a solitary tumor gland surrounded by adipose tissue, was sufficient to classify the case as SARIFA positive. Experienced pathologists (NGR; BM) blinded to clinical data, clinical course, and other tumor characteristics conducted all tumor evaluations. In rare cases where SARIFA status was unclear, a consensus diagnosis was made through consultation with a second board-certified pathologist using a double-headed microscope. Figure 1 illustrates representative sections of SARIFA-positive and SARIFA-negative CRC samples.

### Analysis of lymphocyte subsets via flow cytometry

Blood samples were obtained prior to surgery, 7–10 days and six months after surgical resection, and flow cytometry was performed within 24 h in our local laboratory using an FC 500 flow cytometer (Beckman Coulter, Brea, CA, USA). The gating strategy for NK cells followed our previously published protocol^4,21^. For cell staining, antibodies from Beckman Coulter and Biolegend (San Diego, CA, USA) were used.

Absolute lymphocyte counts were derived from leukocyte counts measured with Stem-Count (Stem-Kit, Beckman Coulter), while initial lymphocyte values were reported as percentages. NK cells were detected as CD56 positive CD3 negative lymphocytes and further subdivided into three functional subsets: CD56 + CD16+, CD56dim CD16bright, and CD56bright CD16dim. The gating strategy is depicted in the supplementary data (Supplementary Fig. 1).

### Analysis of inflammatory mediators (IM) via multiplex ELISA

IM levels were measured using the multiplex immunoassay CodePlex Secretome^→^ solution for IsoSpark$^\circledR$ technology (Bruker Cellular Analysis, United States) on cryopreserved plasma samples obtained at the same time points as the samples for flow cytometry. The macrochambers of the CodePlex$^\circledR$ chip were pre-patterned with a 22-plex antibody array, containing GM-CSF, GranzymeB, IFN-γ, IL-10, IL13, IL-15, IL-17 A, IL-2, IL-4, IL-5, IL-6, IL-7, IL-8, IL-9, IP-10, MCP-1, MIP-1a, MIP-1b, Perforin, sCD137, TNF-α, TNF-ß (Codeplex Secretome Adaptive Immune panel). Samples were thawed at room temperature and filled into each well of a chip in replicates (5.5 µL per replicate). 2% BSA (Bovine Serum Albumin) diluted in PBS (Phosphate-Buffered Saline) was used for background measurements. The chip was then loaded into an IsoSpark$^\circledR$ system, and IM were measured in bulk using a fluorescence enzyme-linked immunosorbent assay (ELISA)-based multiplex assay in an automated workflow. Results were quantified by IsoSpeak$^\circledR$ software v3.0.1 (Bruker Cellular Analysis, United States). Outputs were averaged between replicates and given in relative fluorescence units (RFU).

### Statistical analysis

Descriptive results were expressed as medians with interquartile ranges. Differences in relative frequencies were evaluated using Fisher’s exact test. For group comparisons between healthy controls, SARIFA-negative and SARIFA-positive cases, global p value with Kruskal-Wallis test was calculated and a post hoc paired Mann-Whitney-U test complemented. Longitudinal values were compared using Friedman test with post hoc Wilcoxon signed-rank test. Given the limited cohort size and the exploratory character of this post hoc analysis, no formal correction for multiple comparisons was applied; accordingly, the comparisons of the 22 inflammatory mediators across groups and time points should be regarded as exploratory and hypothesis-generating, and the corresponding p values as nominal and uncorrected. The significance levels are indicated as follows: * *p* < 0.05, ** *p* < 0.01, *** *p* < 0.001, and **** *p* < 0.0001. All analyses were performed using SPSS for Windows version 24 (IBM, Armonk, NY, USA). Figures were generated using SPSS or Matplotlib (version 3.10.0).

## Results

### Patient demographics and tumor characteristics

The patient cohort with resectable CRC comprised 40 patients with 12 (30%) SARIFA positive patients and 28 (70%) SARIFA negative. The median age was 61 years (range, 46–77) and 68 years (range 48–84), respectively (ns). Sex distribution differed significantly (*p* = 0.005). Women predominated in the SARIFA positive group (75%), whereas men were more frequent in the SARIFA negative group (75%). Tumor stage (UICC I–II, 58.3% vs. 75%), tumor side (right, 66.7% vs. 67.9%), and microsatellite status (MSS, 83.3% vs. 71.4%) did not differ significantly between groups (Table 1). There were no statistically significant differences between the two groups regarding postoperative complications, locoregional recurrence and metastatic disease.

**Table 1:** Demographics, tumor characteristics and complication rate of the patient cohort. Complications are classified according to the Clavien-Dindo classification as published by Dindo et al.^23^ MSI/dMMR, microsatellite-instable/deficient mismatch repair; MSS/pMMR, microsatellite-stable/proficient mismatch-repair; SARIFA, Stroma AReactive Invasion Front Areas; UICC, Union for International Cancer Control.

Variable	SARIFA positive *n* = 12 (30%)	SARIFA negative *n* = 28 (70%)	*p*- value
Age: median (range)	61 (46- 77)	68 (48 - 84)	ns
Gender			**0.005**
male; n (%)	3 (25)	21 (75)	
female; n (%)	9 (75)	7 (25)	
Stage			
UICC I and II; n (%)	7 (58.3)	21 (75)	ns
UICC III; n (%)	5 (41.7)	7 (25)	
Tumor Side			
Right; n (%)	8 (66.7)	19 (67.9)	ns
Left; n (%)	4 (33.3)	9 (32.1)	
Microsatellite status			
MSS (pMMR); n (%)	10 (83.3)	20 (71.4)	ns
MSI (dMMR); n (%)	2 (16.7)	7 (25)	
Information not available		1	
Complications			
no complications; n (%)	6 (50)	19 (67.9)	ns
Clavien-Dindo I and II; n (%)	0	2 (7.1)	
Clavien-Dindo III and IV; n (%)	6 (50)	7 (25)	
Recurrence			
Locoregional recurrence; n (%)	0	1 (3.6)	ns
Metastatic disease; n (%)	2 (16.6)	2 (7)	
Information not available	5	7	

### Flow cytometric analysis of NK cell counts

Total NK cells and three different subsets from the peripheral blood were measured by flow cytometry. Postoperative values were available for 38 patients for the day 7–10 timepoint and for 25 patients for the six-month timepoint. At the six-month time point, paired samples were available for 17 of 28 SARIFA-negative (61%) and 8 of 12 SARIFA-positive patients (67%); the proportion of patients without a six-month sample did not differ significantly between the two groups (*p > 0.99*, Fisher’s exact test). Missing six-month measurements were attributable to the absence of a routine follow-up blood draw or loss to clinical follow-up mainly due to the COVID-19 pandemic rather than to disease-related events, and baseline demographic and tumor characteristics of patients with and without an available six-month sample were comparable (Supplementary Tables 1 and 2).

**Fig. 2:**
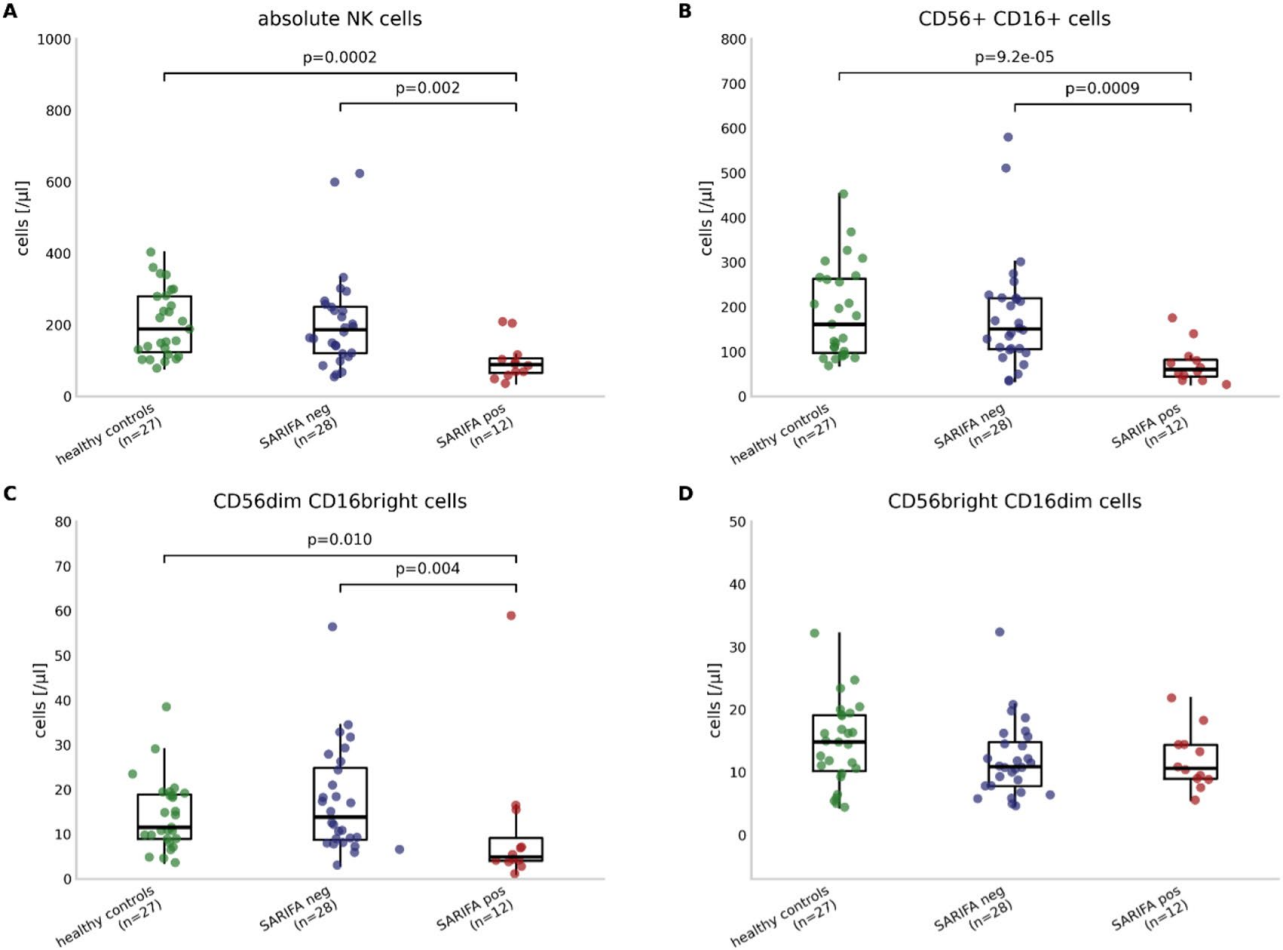
Comparison of NK cells in general **(A)** and CD56 + CD16+ subtype **(B)** as well as between CD56dim CD16bright **(C)** and CD56bright CD16dim **(D)** NK cell subtypes between healthy controls (green), SARIFA-pos(itive) (red) and SARIFA-neg(ative) (blue) CRC patients. The results represent a subgroup analysis (UICC I-III) of previously published data which included stage IV CRCs^15^. NK, Natural Killer cells; SARIFA, Stroma AReactive Invasion Front Areas; UICC Union for International Cancer Control.

As we have previously shown for this cohort for CRC patients of UICC stages I-IV^15^, preoperative total NK-cell counts were markedly reduced in SARIFA-positive patients compared with SARIFA negative (89 cells µl^-1^ vs. 187 cells µl^-1^; *p* = *0.002*) and healthy individuals (89 cells µl^-1^ vs. 226 cells µl^-1^, *p* = 0.0002), when only focusing on UICC stages I-III. Significant differences between the two CRC subgroups were likewise observed within NK-cell subsets, including CD56dimCD16bright and CD56 + CD16+ populations (14 cells µl^-1^ vs. 5 cells µl^-1^; *p* = *0.004* and 151 cells µl^-1^ vs. 61 cells µl^-1^; *p* = 0.0009, respectively). CD56brightCD16dim cells did not differ significantly between the SARIFA-positive and -negative cohort and healthy individuals (11 cells µl^-1^ vs. 11 cells µl^-1^; 11 cells µl^-1^ vs. 15 cells µl^-1^) (Fig. 2).

**Fig. 3:**
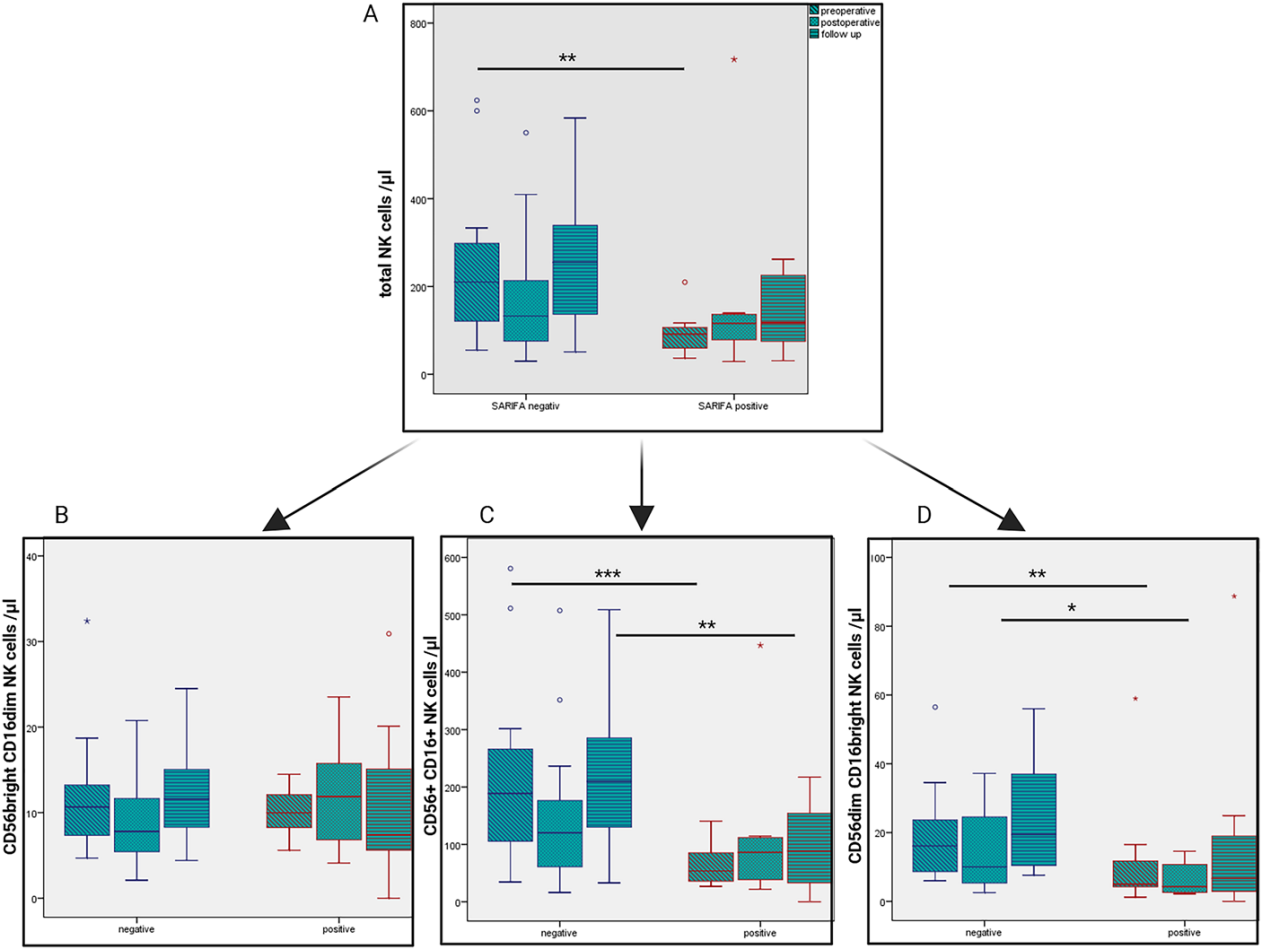
Pre- versus postoperative values of total NK cells **(A)** and their subsets of CD56brightCD16dim **(B)**, CD56 + CD16+ **(C)** and CD56dimCD16bright **(D)** cells at the three distinct time points: preoperative (left boxplot), postoperative (center), and follow-up (right boxplot) for SARIFA negative (blue frame) and SARIFA positive (red frame) patients. Samples were available for 28, 26 and 17 patients at the three timepoints in the SARIFA-negative cohort and for 12, 12 and 8 patients in the SARIFA-positive cohort. * *p* < 0.05, ** *p* < 0.01, *** *p* < 0.001, **** *p* < 0.0001. NK, Natural Killer cells; SARIFA, Stroma AReactive Invasion Front Areas created with biorender.

Figure 3 displays the postoperative course of NK cells in SARIFA-positive and -negative patients. Total NK cells in SARIFA-negative patients significantly decreased after surgery (187 cells µl^-1^ vs. 117 cells µl^-1^; *p* = 0.0004). As well the cellular subsets of CD56dimCD16bright, CD56 + CD16+ and CD56brightCD16dim NK cells showed significant decreases immediately after surgery (14 cells µl^-1^ vs. 9 cells µl^*− 1*^, *p* = 0.009; 150 cells µl^-1^ vs. 68 cells µl^*− 1*^, *p* < 0.001; 11 cells µl^-1^ vs. 8 cells µl^-1^, *p* < 0.0001, respectively) in the SARIFA-negative cohort. In contrast, in SARIFA-positive patients, total NK cell counts remained largely unchanged pre- and postoperatively (89 cells µl^-1^ vs. 93 cells µl^-1^, *p* = 0.158). CD56 + CD16+ cells significantly increased postoperatively in SARIFA-positive patients (61 cells µl^-1^ vs. 81 cells µl^-1^, *p* = 0.002). No statistical difference was observed for CD56dimCD16bright and CD56brightCD16dim cells.

In SARIFA-negative patients the postoperative decrease of NK cells was resolved six months after surgery leading to comparable values as prior to surgery (pre surgery: 187 cell µl^-1^ vs. six months follow up: 251 cells µl^-1^; *p* = 0.227). In SARIFA-positive patients a trend towards higher NK cell values was observed six months after surgery (pre surgery: 89 cells µl^-1^ vs. six months follow up: 117 cells µl^-1^, *p* = 0.063). However, at the follow-up measurements a significant difference remained for the largest subgroup of CD56 + CD16+ NK cells between SARIFA-positive and SARIFA-negative patients (88 cells µl^-1^ vs. 214 cells µl^-1^; *p* = 0.007). Total NK cells and CD56dimCD16bright NK cells showed a trend towards lower values in SARIFA positive patients at the six months follow up, not reaching statistical significance (117 cells µl^-1^ vs. 251 cells µl^-1^; *p =* 0*.065* and 7 cells µl^-1^ vs. 19 cells µl^-1^; *p* = 0.057 respectively).

### Inflammatory mediatiors

**Fig. 4:**
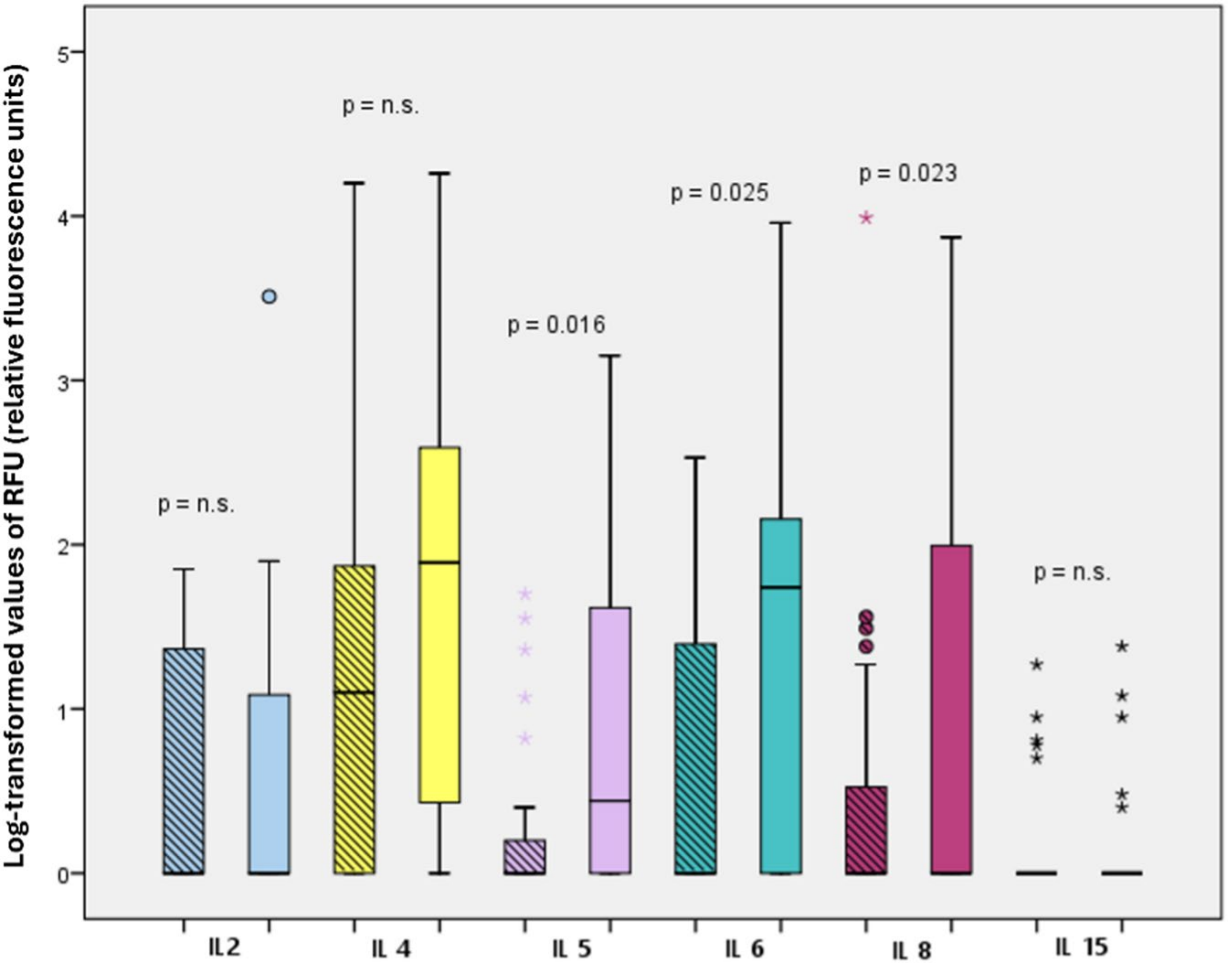
Representative examples of inflammatory mediators for the whole CRC-cohort measured preoperatively and 7–10 days postoperatively. The data for each interleukin are represented in boxplots, comparing log-transformed values of the relative fluorescence units (RFU) of six interleukins: IL 2, IL 4, IL 5, IL 6, IL 8, and IL 15. IL=interleukin.Preoperative measurements are depicted as shaded on the left side of each boxplot; clear boxplots represent values postoperatively. No formal correction for multiple comparisons was applied, comparisons of the 22 inflammatory mediators are exploratory and hypothesis-generating with nominal, uncorrected p values.

To detect potential differences in inflammatory mediators that might be associated with the alterations in NK cells, multiplex ELISA using a predefined antibody panel was used on pre- and postoperative plasma samples. We observed significant differences in IL-5, IL-6 and IL-8 levels in the total CRC-cohort regardless of SARIFA status pre- vs. post surgery as depicted in Fig. 4. Significant differences pre- and postoperatively were likewise observed for IL-4, IL-5, IL-6, IL-8 and sCD137 in the SARIFA negative cohort (*p =* 0.028, *p =* 0.013, *p =* 0.026, *p =* 0.038 and *p =* 0.046, respectively). In the SARIFA positive cohort, only perforin differed significantly at the two timepoints (*p =* 0.028). As these comparisons were not corrected for multiple testing, the isolated nominal differences clustering between *p* = 0.028 and *p* = 0.046—in particular the within-group perforin change in SARIFA-positive patients—may in part represent false-positive findings and should be regarded as exploratory.

Regarding differences in interleukine levels between SARIFA-negative and -positive patients pre- and postoperatively, we did not observe significantly different levels of inflammatory mediators except for lower IL-4 in SARIFA-positive patients compared with SARIFA-negative patients (*p = 0.029*) at the postoperative timepoint (Supplementary Fig. 2). As this isolated nominal difference was not corrected for the large number of analytes and comparisons performed, it may represent a false-positive result and is reported as an exploratory observation only.

## Discussion

In our previous study on this cohort, we could show that NK cells are reduced in SARIFA-positive CRC patients not only in the peripheral blood but also at tissue level^15^. A large Finnish study could validate the immune dysregulation of SARIFA-positive CRCs at the tissue level^5^. We now sought to investigate the dynamics of NK cell populations and immune modulators, like interleukines, not only at the pre-operative stage but also in the postoperative course of disease, as this has not been studied in detail before.

By doing so, we observed significant differences in the NK cell populations between SARIFA-negative and SARIFA-positive colorectal cancer patients at different pre- and postoperative time points. These differences provide important and additional insights into the immune landscape of CRC patients and the potential relation between SARIFA and the immune system.

Initially, preoperative NK cell counts in SARIFA-negative patients were comparable to those found in the general, healthy population. In contrast, SARIFA-positive patients exhibited lower NK cell counts, suggesting that either the presence of a SARIFA-positive tumor exerts an immunosuppressive effect leading to lower NK cells, or lower NK cells as a patient-intrinsic factor are associated with the development of SARIFA-positive tumors.

Postoperatively, we observed a clear decrease in NK cell numbers in SARIFA-negative patients. Reduced numbers of NK cells after surgery in the blood of colorectal cancer patients have been reported before and were attributed to the perioperative stress and surgical trauma associated with colon and rectum resections^24,25^. Additionally, functional alterations with reduced NK cell activity and cytotoxicity seem to be relevant and have been addressed in different studies^26,27^. This functional aspect cannot be answered in our study as a further characterization of functional NK cell markers was not performed and will be addressed in further investigations. The question why NK cells decline after surgery only in SARIFA-negative patients is not easy to answer. Despite the perioperative immunosuppressive effects, NK cells in SARIFA-positive patients remained stable at their lower level. This might suggest that the removal of the SARIFA positive tumor, which may have exerted an immunosuppressive effect, could have had a greater impact on NK cell recovery than the postoperative immunosuppression. An alternative explanation is that NK cell levels in SARIFA-positive patients were already markedly reduced at baseline, potentially limiting the detectable magnitude of further perioperative decline and creating a floor effect that masks additional postoperative suppression. A direct effect of the local tumor microenvironment on NK cells has been described in various publications, indicating functional alterations induced by the tumor itself^28,29^. However, absolute numbers of NK cells were not in the focus of these studies.

At six months follow-up, the persisting differences in NK cell counts between SARIFA-positive and -negative patients indicate that tumor-derived immunosuppression alone is unlikely to fully explain the observed immune alterations. To exclude bias due to local tumor recurrence or metastasis, all patients were checked for recurrence and no difference between the SARIFA-positive and negative cohort was observed within one year after surgery. This finding is consistent with the hypothesis that a patient-intrinsic immune dysregulation, rather than a sustained tumor-derived effect, is associated with the development of SARIFA-positive tumors, since a prolonged, dominant immunosuppressive effect of the primary tumor persisting for months after resection would appear less likely. However, because this is an observational post hoc analysis that did not adjust for potential confounders (see below), these data establish an association between SARIFA status and persistently reduced NK cell counts but cannot establish that patient-intrinsic immunosuppression causally contributes to the development of SARIFA-positive tumors.

To determine whether the observed NK cell dynamics were influenced by changes in interleukin levels, we measured concentrations of multiple inflammatory mediators pre- and postoperatively in both patient cohorts. Interleukins play central roles in immune regulation, with distinct cytokines modulating inflammatory and immune responses during surgery and recovery. IL-2 is essential for T-cell activation, whereas IL-4, IL-5, and IL-6 contribute to the regulation of immune and inflammatory pathways. IL-8 mediates neutrophil recruitment to sites of tissue injury and IL-15 supports NK cell survival and activation^30–34^. The postoperative alterations in interleukin levels were most likely attributable to perioperative acute inflammatory responses. Moreover, given the highly dynamic nature of interleukins, variations may also reflect differences in the exact timing of sample collection. Relevant differences in cytokine levels between SARIFA-positive and -negative patients could not be observed. These largely negative results temper the proposed adipocyte–cytokine–NK-cell axis as a systemic explanation for our findings: despite the persistently reduced NK cell counts in SARIFA-positive patients, we did not detect consistent between-group differences in IL-6, IL-8 or the other measured mediators that would be expected if circulating adipocyte- or tumor-derived cytokines were the principal driver of NK cell suppression. The interactions underlying this axis have been demonstrated mainly at the local tumor–adipocyte interface and in experimental co-culture and murine models and may therefore operate predominantly within the tumor microenvironment without being mirrored in peripheral cytokine concentrations. Equally, the predefined panel, the single perioperative sampling windows and the small cohort may have precluded the detection of subtle systemic differences. The proposed mechanism should therefore be regarded as a hypothesis that our cytokine data do not, on their own, support.

A major strength of this study is the direct integration of a histopathological invasion front biomarker (SARIFA) with longitudinal, peripheral blood immune profiling, enabling the linkage of localized tumor-stroma interactions to systemic immune dynamics, an approach that is rarely undertaken in CRC research, and has only recently been explored for other histopathological features such as tumor necrosis, which was shown to associate with systemic cytokine alterations and adverse prognosis^35^. The prospective sampling at multiple perioperative time points further allowed the temporal dissection of tumor-related versus surgery-induced immune alterations. However, several limitations should be acknowledged. First, this was an observational post hoc analysis in which the two groups were not balanced for all baseline characteristics. In particular, sex distribution differed significantly between the SARIFA-positive and SARIFA-negative groups (*p = 0.005*; women comprising 75% of the SARIFA-positive and 25% of the SARIFA-negative group). Because peripheral NK cell counts and subset composition are known to vary with sex and age, this imbalance represents a potential confounder for the central NK cell comparison and cannot be fully excluded as a contributor to the observed differences. Given the limited cohort size, robust multivariable adjustment was not feasible; while the consistency of the pattern across time points and the persistence of the CD56 + CD16+ difference argue against sex being the sole explanation, the findings should be interpreted with this imbalance in mind. In the initial publication of the total cohort^4^ a multivariate analyse including age and sex was included and no significant alterations of total NK cells depending on sex were seen. In addition, several further factors known to influence NK cell counts and function—including obesity and visceral adiposity, diabetes mellitus, nutritional status, and concomitant medication—were not systematically recorded and could not be adjusted for. Our data therefore support an association between SARIFA status and persistently reduced NK cell counts but do not prove that a patient-intrinsic immunosuppression causally drives the development of SARIFA-positive tumors. Moreover, the principal novel finding—the persistence of the difference at six months—rests on small numbers (17 SARIFA-negative versus 8 SARIFA-positive patients), and only the CD56 + CD16+ subset reached statistical significance (*p = 0.007*), whereas total NK cells and CD56dimCD16bright cells reached only trend level (*p = 0.065* and *p* = 0.057); these observations require confirmation in larger, prospectively balanced cohorts. Functional characterization of NK cell activity was not performed, precluding direct conclusions on cytotoxic capacity beyond cell abundance. In addition, inflammatory mediator measurements were restricted to predefined panels and single perioperative windows, which may not fully capture the dynamic cytokine landscape. Future studies with larger cohorts, functional immune assays, and extended longitudinal sampling will be required to further delineate the mechanistic basis of SARIFA-associated immune dysregulation.

In conclusion, we showed persisting differences in NK cell values in the peripheral blood of SARIFA-positive CRC patients that are associated with this aggressive tumor phenotype and that may contribute to the poorer prognosis of SARIFA positive tumors. Given the observational design, the limited cohort and the predominantly trend-level differences at six months, these results are hypothesis-generating and require validation in larger, prospectively balanced cohorts before causal or therapeutic implications can be drawn. Our findings emphasize the need for further investigation into potential therapeutic strategies aimed at reversing the immune suppression associated with SARIFA-positive tumors.

## Supplementary Information

Below is the link to the electronic supplementary material.

Supplementary Material 1

Supplementary Material 2

Supplementary Material 3

## Data Availability

The datasets generated and/or analyzed during the current study are available from the corresponding author on reasonable request—and to some parts only in restricted form for privacy reasons.
