# Supplementary figures and images for: Distinct NK cell dynamics in SARIFA positive colorectal cancer patients indicate persistent patient-intrinsic immune signatures after tumor resection
Source: Sci Rep. 2026 Jul 29;16:23536. doi: 10.1038/s41598-026-63238-z (PMC13416078; doi:10.1038/s41598-026-63238-z)

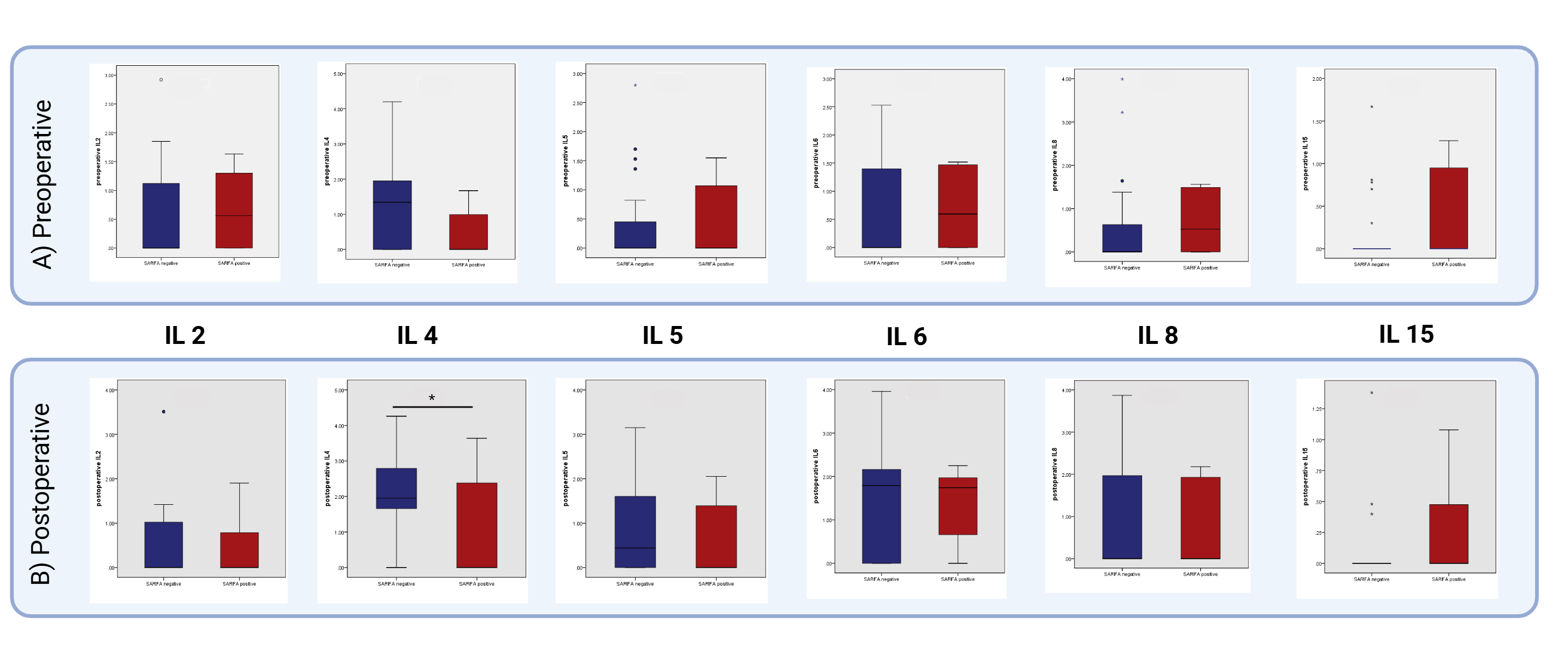

Supplement: Supplementary file 1 — Supplementary Material 1 [file 41598_2026_63238_MOESM1_ESM.png]

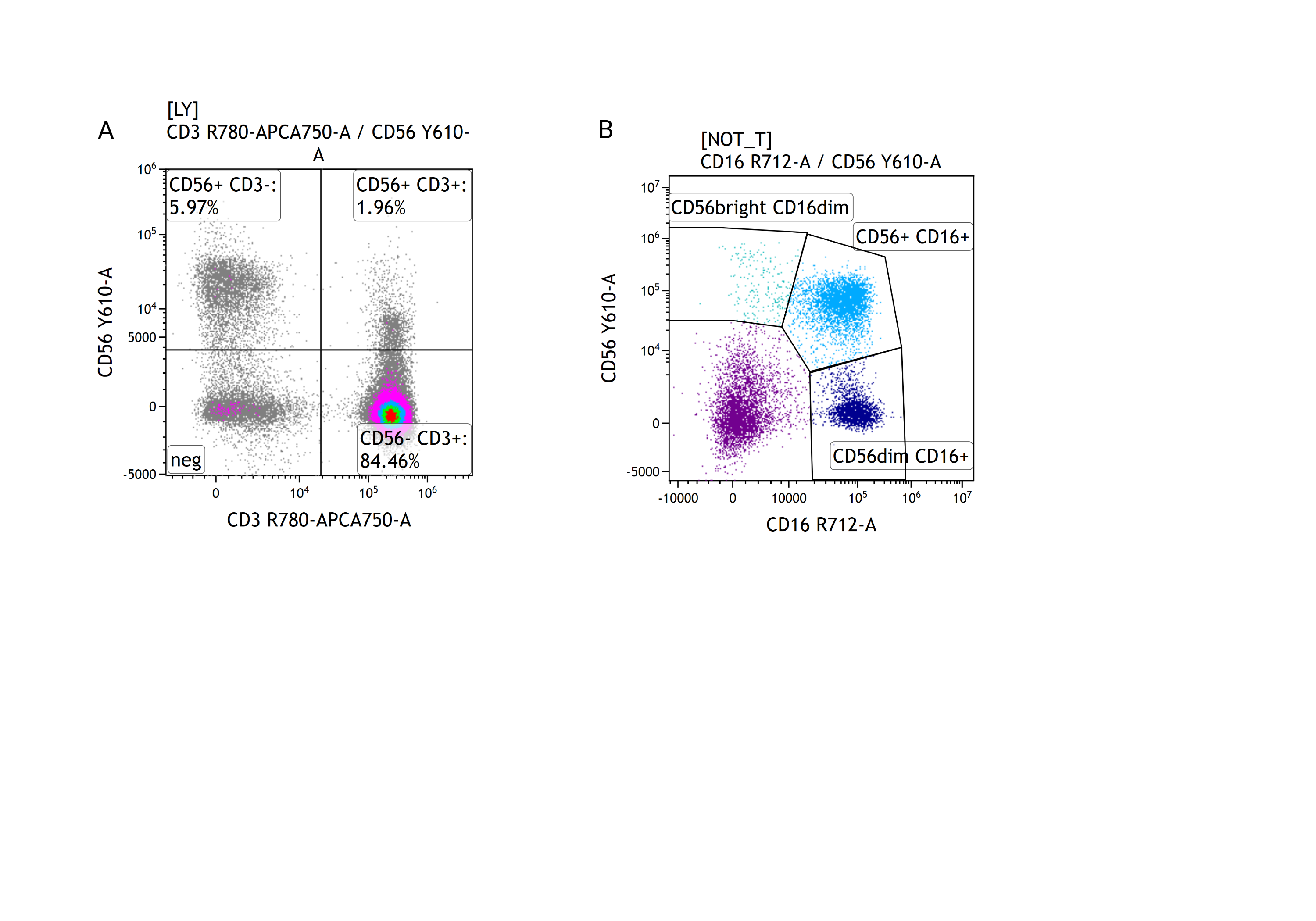

Supplement: Supplementary file 2 — Supplementary Material 2 [file 41598_2026_63238_MOESM2_ESM.png]
