# Supplementary material for: Distinct NK cell dynamics in SARIFA positive colorectal cancer patients indicate persistent patient-intrinsic immune signatures after tumor resection
Source: Sci Rep. 2026 Jul 29;16:23536. doi: 10.1038/s41598-026-63238-z (PMC13416078; doi:10.1038/s41598-026-63238-z)
Supplement: Supplementary file 3 — Supplementary Material 3 [file 41598_2026_63238_MOESM3_ESM.pdf]

Supplementary Figures and Tables:

Supplementary Figure 1: Gating strategy for NK cells

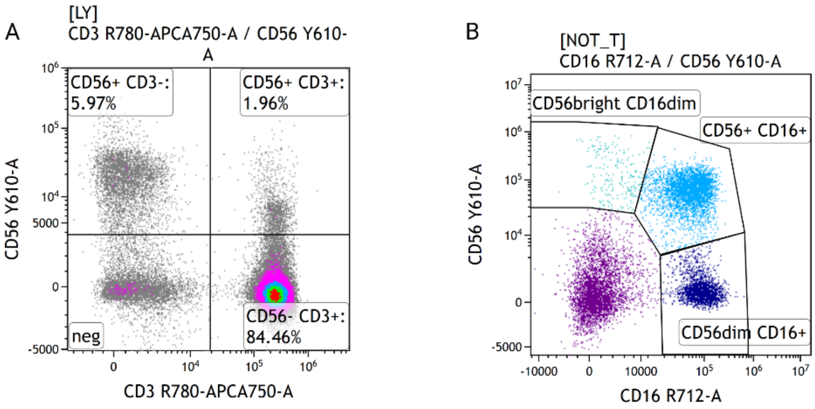

Figure S1: Gating strategy of A) total NK cells, defined as CD56+CD3- lymphocytes and B) NK cell subsets, defined by CD56+CD16+ lymphocytes excluding t cells and subdivided into three subgroups of CD56brightCD16dim, CD56+CD16+ and CD56dimCD16bright cells.

Supplementary Figure 2: Pre- and postoperative Interleukins

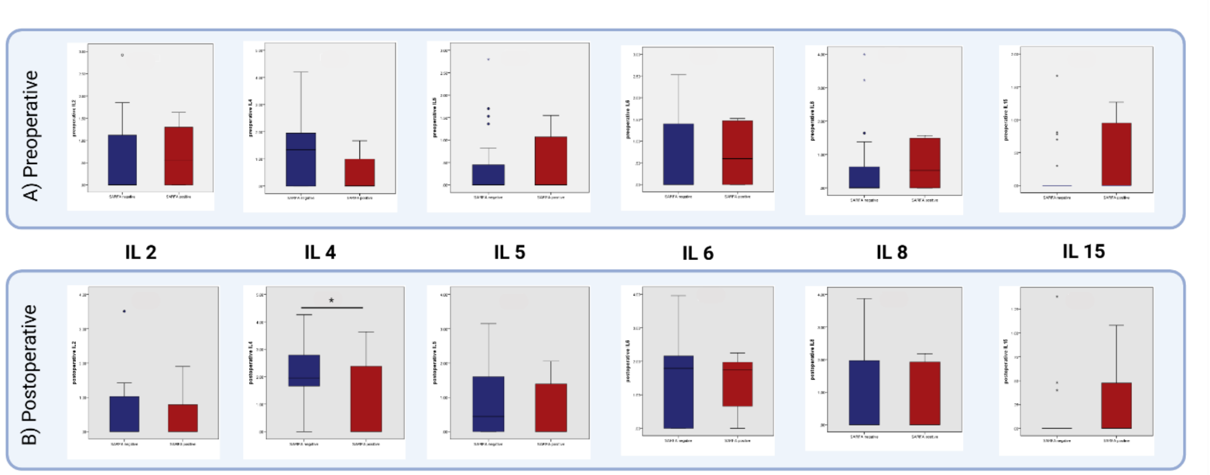

Figure S2: *A) preoperative and B) postoperative (7 – 10 days) values of six key interleukins (IL 2, IL 4, IL 5, IL 6, IL 8, and IL 15) compared between SARIFA-negative (blue) and SARIFA-positive (red) patients. * $p < 0.05$, ** $p < 0.01$, *** $p < 0.001$, **** $p < 0.0001$.*

Supplementary Table 1: Baseline characteristics of patients without a six-month follow-up sample (non-completers), by SARIFA status

Variable	SARIFA positive n = 5	SARIFA negative n = 10	p-value
**Age: median (range)**	66 (49–77)	66 (61–83)	ns
**Gender**			ns
male; n (%)	2 (40)	6 (60)	
female; n (%)	3 (60)	4 (40)	
**Stage**			ns
UICC I and II; n (%)	3 (60)	8 (80)	
UICC III; n (%)	2 (40)	2 (20)	
**Tumor side**			ns
right; n (%)	3 (60)	7 (70)	
left; n (%)	2 (40)	3 (30)	
**Microsatellite status**			n.t.
MSS (pMMR); n (%)	5 (100)	8 (80)	
MSI (dMMR); n (%)	0 (0)	0 (0)	
information not available; n (%)	0 (0)	2 (20)	
**Complications**			ns
no complications; n (%)	2 (40)	6 (60)	
Clavien-Dindo I and II; n (%)	0 (0)	1 (10)	
Clavien-Dindo III and IV; n (%)	3 (60)	3 (30)	
information not available; n (%)	0 (0)	0 (0)	
**Recurrence**			n.t. ¶
no recurrence; n (%)	0 (0)	3 (30)	
locoregional recurrence; n (%)	0 (0)	0 (0)	
metastatic disease; n (%)	1 (20)	2 (20)	
information not available; n (%)	4 (80)	5 (50)	

Statistical analysis: Fisher's exact test (two-sided) for categorical variables; α = 0.05. Percentages refer to the column total (n = 5 or n = 10) and were recomputed accordingly. Categories "information not available" were treated as missing and excluded from the corresponding test. None of the comparisons reached statistical significance.
Ns= not significant; nt= not tested

Supplementary Table 2: Comparison of baseline characteristics between patients with (completers) and without (non-completers) a six-month follow-up sample

Variable	Completers n = 25	Non-completers n = 15	p-value
**Age: median**	66	66	ns
**Gender**			ns
male; n (%)	16 (64)	8 (53)	
female; n (%)	9 (36)	7 (47)	
**SARIFA status**			ns
SARIFA-positive; n (%)	7 (28)	5 (33)	
SARIFA-negative; n (%)	18 (72)	10 (67)	
**Tumor side**			ns
right; n (%)	17 (68)	10 (67)	
left; n (%)	8 (32)	5 (33)	
**Microsatellite status**			ns
MSS (pMMR); n (%)	17 (68)	13 (87)	
MSI (dMMR); n (%)	8 (32)	1 (7)	
information not available; n (%)	0 (0)	1 (7)	
**Complications**			ns
no complications; n (%)	17 (68)	8 (53)	
Clavien-Dindo I and II; n (%)	1 (4)	1 (7)	
Clavien-Dindo III and IV; n (%)	7 (28)	6 (40)	

Statistical analysis: Fisher's exact test (two-sided) for categorical variables; α = 0.05. "Information not available" was treated as missing and excluded from the corresponding test. None of the comparisons reached statistical significance, indicating no detectable selection bias between patients with and without a six-month sample.
